# Mechanisms underlying skeletal muscle insulin resistance induced by fatty acids: importance of the mitochondrial function

**DOI:** 10.1186/1476-511X-11-30

**Published:** 2012-02-23

**Authors:** Amanda R Martins, Renato T Nachbar, Renata Gorjao, Marco A Vinolo, William T Festuccia, Rafael H Lambertucci, Maria F Cury-Boaventura, Leonardo R Silveira, Rui Curi, Sandro M Hirabara

**Affiliations:** 1 Department of Physiology and Biophysics, Institute of Biomedical Sciences, University of São Paulo, Av. Professor Lineu Prestes, 1524, Butantã, São Paulo 05508-000, SP, Brazil; 2 Post-Graduate Program in Human Movement Sciences, Institute of Physical Activity Sciences and Sports, Cruzeiro do Sul University, São Paulo, SP, Brazil

**Keywords:** Skeletal muscle, Insulin resistance, Saturated fatty acids, Mitochondrial dysfunction

## Abstract

Insulin resistance condition is associated to the development of several syndromes, such as obesity, type 2 *diabetes mellitus *and metabolic syndrome. Although the factors linking insulin resistance to these syndromes are not precisely defined yet, evidence suggests that the elevated plasma free fatty acid (FFA) level plays an important role in the development of skeletal muscle insulin resistance. Accordantly, *in vivo *and *in vitro *exposure of skeletal muscle and myocytes to physiological concentrations of saturated fatty acids is associated with insulin resistance condition. Several mechanisms have been postulated to account for fatty acids-induced muscle insulin resistance, including Randle cycle, oxidative stress, inflammation and mitochondrial dysfunction. Here we reviewed experimental evidence supporting the involvement of each of these propositions in the development of skeletal muscle insulin resistance induced by saturated fatty acids and propose an integrative model placing mitochondrial dysfunction as an important and common factor to the other mechanisms.

## Introduction

Insulin resistance is broadly defined as the reduction in insulin ability to stimulate glucose uptake from body peripheral tissues. At physiological conditions, insulin activates glucose uptake by stimulating the canonical IRS-PI3K-Akt pathway and by phosphorylating and inactivating Akt substrate 160 (AS160), a protein that, when activated, prevents glucose transporter (GLUT) 4 translocation to the membrane. Thus, by inhibiting AS160, insulin promotes the GLUT4 translocation from inner vesicules, promoting fusion to the plasma membrane and consequently glucose uptake [1].

Although insulin resistance is a key component of several chronic syndromes associated with obesity such as type 2 *diabetes mellitus *and metabolic syndrome, the involved factors and their underlying mechanisms linking excessive adiposity to insulin resistance were not completely elucidated yet [2-5]. Evidence suggests that fatty acids, whose circulating levels are markedly increased in obesity and associated-diseases, might play a role in the development of skeletal muscle insulin resistance [6,7]. In this sense, prolonged exposure of skeletal muscle and myocytes to high levels of fatty acids leads to severe insulin resistance [8,9]. Among the different types of fatty acids, saturated long-chain fatty acids such as palmitic and stearic acids were demonstrated to be potent inducers of insulin resistance [5,10]. Several mechanisms have been suggested by us [2,5,11,12] and others [6,8,13-16] to explain how saturated fatty acids impair insulin actions such as the Randle cycle, accumulation of intracellular lipid derivatives (diacylglycerol and ceramides), oxidative stress, modulation of gene transcription, inflammation and mitochondrial dysfunction. In the present review, we discuss evidence supporting the involvement of these mechanisms in the regulation of insulin sensitivity by saturated fatty acids and propose the mitochondrial dysfunction found in conditions of elevated fatty acid levels has a central role in the pathogenesis of insulin resistance.

### Mechanisms underlying the insulin resistance induced by saturated fatty acids

#### Competition between fatty acids and glucose: the randle cycle

The first mechanistic explanation for the inverse relationship between fatty acids availability and glucose utilization was proposed by Randle *et al. *[13]. In this study, it was shown that an elevation in fatty acids supply to diaphragm and isolated heart is associated with an increase in fatty acid oxidation and an impairment in glycolytic flux and glucose utilization, such effect being mediated by alosteric inhibition of glycolytic enzymes. More specifically, the proposed hypothesis was that increased fatty acid oxidation raises the production of acetyl-CoA resulting in inhibition of pyruvate dehydrogenase activity and elevation of citrate levels at the tricarboxylic acid cycle. Citrate together with an increased ATP/ADP ratio reduce the activity of phosphofructokinase and consequently glucose flux through the glycolytic pathway, resulting in glucose 6-phosphate accumulation, hexokinase II inhibition, increase in intracellular glucose content and, consequently, reduction in glucose uptake [17,18].

In accordance with Randle's hypothesis, elevation in circulating fatty acids levels by either intralipid/heparin or lipid infusion in rats, humans and type 2 *diabetes mellitus *patients is associated with impairments in glucose uptake, utilization and oxidation in insulin-sensitive tissues (heart, skeletal muscle and adipose tissue) [19-21]. Acutely, fatty acids lead to Randle cycle effect, increasing intracellular content of citrate and glucose-6-phosphate and decreasing glycolytic pathway flux [2,11]. It has been also demonstrated that palmitate accutely increases glucose uptake in L6 myotubes by activating insulin signaling pathways (Akt and ERK1/2) [22].

However, in contrast to Randle's hypothesis, in which intracellular glucose accumulation must precede the inhibition of glucose uptake, further studies demonstrated that the insulin resistance induced by fatty acids is primarily associated with impaired glucose uptake rather than changes in hexose metabolism [8,18]. In studies involving lipid infusion associated with other techniques including glucose and insulin clamp and nuclear magnetic resonance a rapid reduction in glycolysis (previous to 2 hours) followed by impaired glucose disposal and glycogen synthesis (between 4-6 hours) was observed [7,14]. Roden *et al. *[14] demonstrated that the reduction in muscle glycogen synthesis is preceded by a decrease in intramuscular glucose 6-phosphate, suggesting that the increase in plasma fatty acid concentration initially induces insulin resistance by inhibiting glucose transport or its phosphorylation. Other studies also demonstrated that lipid infusion decreases intracellular glucose and glucose 6-phosphate content, due to inhibition of glucose uptake by skeletal muscle [8,23]. These studies demonstrated that Randle cycle does not completely explain the effects of FFA on glucose metabolism indicating that other mechanisms are also involved in the FFA-induced insulin resistance.

### Inhibition of skeletal muscle insulin signaling by saturated fatty acids

In addition to its important effects on glucose metabolism directly, saturated fatty acids were demonstrated to affect insulin intracellular signaling pathways in skeletal muscle and myocytes [5,14,24,25]. Studies have demonstrated a marked reduction in IRS-1 tyrosine phosphorylation [9], IRS-1 and -2-associated PI3-kinase activity [9,26], and Akt phosphorylation and activity [26] in skeletal muscle after lipid infusion in euglycemic-hyperinsulinemic clamp. Along with a direct effect of saturated fatty acids on skeletal muscle insulin signaling, palmitic acid was shown to decrease insulin receptor expression and activity [27] and phosphorylation of IRS-1 and -2 at tyrosine residues [2,28], Akt [5,28-31] and GSK-3, as reviewed by Schmitz-Peiffer *et al. *[32] in isolated soleus muscle, primary culture of rat myocytes, pmi28 myotubes, C2C12 and L6 myocytes. Similarly to skeletal muscle, palmitic acid inhibits Akt phosphorylation and activity in rat perfused heart and in HL-1 cells, an immortalized cardiomyocyte like cell lineage [33].

Several mechanisms have been postulated to account for the inhibition of insulin signaling by saturated fatty acids, including the activation of various kinases such as PKCs, IKK β, JNK, and p38 MAP kinase. These kinases have been postulated to catalyze the phosphorylation of serine residues in IRS-1 inhibiting its activity and directing it for degradation by the proteasome [34,35]. Such effects culminate with a reduction in the phosphorylation of tyrosine residues of IRS-1 by insulin, blocking its downstream signal transduction [36-38]. Other serine/threonine kinase activated in high-fat diet-induced or palmitate-induced insulin resistance is mammalian target of rapamycin (mTOR) [39,40], but the mechanisms involved are unknown yet.

### Lipotoxic intramyocellular lipid accumulation induced by fatty acids

When the amount of circulating lipids chronically exceeds white adipose tissue ability for uptake and storage, like obesity, fatty acids accumulate in other tissues with limited capacity for lipid storage such as liver and skeletal muscles. Such abnormal ectopic lipid accumulation (lipotoxicity) is strongly associated with insulin resistance [41,42].

Fatty acids accumulate intracellularly in myocytes mainly as long-chain fatty acyl-CoA, monoacylglcyerol, diacylglycerol, phosphatidic acid, triacylglycerol and ceramides [32,43-46]. Among these fatty acid derivatives, high intramyocellular levels of diacylglycerol, triacylglycerol, and ceramides are directly associated with insulin resistance. Corroborating with this hypothesis, high fat feeding is associated with an increase in intramyocellular content of diacylglycerol and triacylglycerol and insulin resistance, such effects being abolished by inhibition of muscle lipid accumulation due to genetic deletion of lipoprotein lipase, fatty acid transporters (CD36 and FATP1), and diacylglycerol acyl transferase-1 (DGAT-1) [47-49]. Diacylglycerol accumulation is associated with the activation of subgroup of novel kinases, members of the large protein kinase C (PKC) family. Among the novel kinases, diacylglycerol directly activates PKCθ that catalyzes the phosphorylation of serine-307 residue at IRS-1, reducing its tyrosine phosphorylation and activation by insulin.

Consistent with this, Schmitz-Peiffer *et al. *[50] reported increased concentration of DAG in rodent's muscle and activation of PKCs induced by high-fat diet. Similarly, infusion of lipid and heparin caused insulin resistance in muscles that was associated with accumulation of intracellular DAG and specific activation of PKCθ [8]. Insulin resistance in this model was due to lipid-induced defects in the insulin signaling pathway that was caused by a reduction in tyrosine phosphorylation of IRS1, increasing its phosphorylation in serine-307 residue [9]. However, there are still no evidence to explain how the activation of novel PKCs might relate to serine phosphorylation of IRS1, and which kinases might have a role in the pathway, as reviewed by Samuel *et al. *[51].

In addition to diacylglycerol accumulation, high-fat diet or palmitate treatment increases production of ceramide and sphingosines in skeletal muscle cells, which is associated with glucose intolerance and insulin resistance [52,53]. Evidence suggests that ceramide and phosphatidic acid mediate the deleterious effects of palmitic acid on insulin signaling in cultured myotubes and insulin mediated Akt and GSK phosphorylation in C2C12 myotubes [52,54]. Ceramides also affects insulin signaling by two distinct mechanisms involving the activation of Akt dephosphorylation at threonine 308 and inhibition of its translocation to the plasma membrane [55], such effects being dependent on ceramides activation of protein phosphatase 2A (PP2A) and PKCζ, respectively [56,57]. In addition, glycosylceramide, a glycosyl derivative of ceramides was shown to inhibit insulin receptor activity inducing insulin resistance [56,57]. Recently, it has been demonstrated that increased lysophosphatidylcholine content, a phosphatidic acid, in L6 myotubes treated with palmitate also leads to JNK activation and IRS-1 Ser307 phosphorylation, contributing to the development of muscle insulin resistance [58].

### Activation of inflammatory signaling pathways by saturated fatty acids

Saturated fatty acids activate inflammatory signaling pathways directly through interaction with members of Toll-like receptor (TLR) family and indirectly through the secretion of cytokines including TNF-α, IL-1β and IL-6 [59-61]. TLRs are an evolutionarily ancient pattern-recognition class of receptors that facilitate the detection of microbes. Saturated fatty acids activate TLR-4 in skeletal muscle promoting c-Jun NH(2)-terminal kinase (JNK) and Iκb kinase (IKK) complex activation, which results in degradation of the inhibitor of κB (IκBα) and nuclear factor-κB (NFκB) activation. Activation of JNK and IKKβ by saturated fatty acids is associated with a marked inhibition of insulin action due to the phosphorylation of serine residues on the insulin IRS-1 and inhibition of its stimulatory phosphorylation of tyrosine residues by the insulin receptor [62,63]. Corroborating with an important contribution of TLR-4 to muscle insulin resistance, mice containing a loss of function by mutation of this receptor are partially protected from fat-induced inflammation and insulin resistance [64]. In addition, diabetic and obese mice have increased skeletal muscle IKK and JNK activities, whose pharmacological and genetic inhibition leads to an improvement in insulin sensitivity and glucose tolerance [65-67].

Coletta and Mandarino [68] demonstrated that changes in genes and proteins from inflammatory pathway contribute to the mitochondrial dysfunction observed in insulin resistant muscle and it can lead to decreased fat oxidation, ectopic fat accumulation, insulin signaling abnormalities and finally insulin resistance. These authors indicate that this mechanism is compatible with and complementary to other hypotheses regarding the vicious cycle connecting inflammation, mitochondrial changes, lipid accumulation and insulin signaling defects. The novel aspect of this mechanism is that it connects inflammatory processes with changes in insulin sensitivity by means of altered mechanosignal transduction due to fibrotic changes.

Fatty acids were also demonstrated to reduce mitochondrial function through induction of pro-inflammatory cytokines. Saturated fatty acids, palmitic and stearic acids, stimulate the secretion of TNF-α, IL-1β and IL-6 in human leukocytes [60,69]. Wen *et al. *[61] showed increased IL-1β production and release by palmitic acid in macrophages through activation of the NLRP3-ASC inflammasome. The pro-inflammatory cytokines have been associated to impaired mitochondrial function, establishing a link between fatty acids and mitochondrial dysfunction. Indeed, some studies reported reduced mitochondrial function in cells exposed to TNF-α, IL-1β or IL-6 [70,71].

### Alteration in gene expression by saturated fatty acids

Evidence has been obtained that fatty acids modulate expression of genes involved in glucose and lipid metabolism. Schmid *et al. *[72] demonstrated that C57BL/6 mice submitted to high-fat diet present reduced expression of enolase, a glycolytic enzyme, and ATP synthase in skeletal muscle. In addition, other enzymes of the glycolytic pathway have been shown to be modulated by fatty acids, such as pyruvate dehydrogenase kinase isozyme 1 (PDK-1), whose expression is increased in pancreatic islets incubated with saturated fatty acids [73], and lactate dehydrogenase A (LDHA), which was downregulated in white adipose tissue from high-fat-fed animals [74]. Moreover, increased intramyocellular lipid content has been associated with down-regulation of PGC-1α and of other genes encoding protein mitochondrial respiratory complexes I, II, III, and IV [75], resulting in impaired mitochondrial biogenesis and function [76]. Some studies identified transcription factors that recognize conserved motifs at the promoters of mitochondrial oxidative phosphorylation genes, such as nuclear respiratory factor (NRF)-1 and GA-binding protein (GABP) (also known as NRF-2) [77]. Studies showed that the peroxisome proliferator activator receptors (PPARs) control mitochondrial gene subsets, modulating fatty acid oxidation (FAO) and uncoupling [77,78]. Later, studies showed that PGC-1α is a transcriptional coactivator of NRF-1, GABP, and PPARs, demonstrating the ability of PGC-1α to integrate physiological signals and to increase mitochondrial biogenesis and oxidative function [79,80]. Thus, a reduction of PGC-1α content in conditions of high fatty acid levels might be associated with impairment of mitochondrial function [5,76]. In addition, insulin-resistant subjects have reduced expression of mitochondrial genes, such as cytochrome c oxidase and complexes I and III subunits of the electron transport chain [81]. Activities of carnitine palmitoyltransferase-1 (CPT-1) and other key mitochondrial enzymes, such as citrate synthase and β-hydroxyacyl-CoA dehydrogenase, have also been found decreased in skeletal muscle from obese and type 2 diabetic individuals [26,82,83]. These changes in gene expression and enzyme activities induced by fatty acids contribute to the reduced mitochondrial oxidative capacity consequently leading to mitochondrial dysfunction.

### Increase in reactive oxygen species by saturated fatty acids

Type 2 *diabetes mellitus*, obesity and the metabolic syndrome are strongly correlated with increased skeletal muscle content of reactive oxygen species (ROS) [84-86]. All conditions cited above contribute to an oxidative environment, modulating insulin sensitivity either by increasing insulin signaling or impairing glucose tolerance. The mechanisms by which this occurs are often multifactorial and complex, involving several cell signaling pathways [87].

Production of ROS can occur in response to diverse *stimuli *including: (1) intracellular factors, such as nutrient metabolism, endoplasmic reticulum stress, and detoxification of various xenobiotics; (2) extracellular factors like signaling through plasma membrane receptors, such as hormones and growth factors and by pro-inflammatory cytokines; and (3) physical-environmental factors (e.g. ultraviolet irradiation) [88-91]. When moderately produced, ROS are involved in important physiological processes that lead to desired cellular responses. However, high ROS production is negatively associated with different biological signaling pathways [87]. ROS can react with multiple cellular components, such as proteins, lipids and nucleic acids, generating reversible or irreversible oxidative modifications. Pathophysiological processes mediated by ROS are more likely to induce irreversible modifications in cellular components, a reasonable definition of the term oxidative stress [88].

Control of vascular tone, cell adhesion, immune responses, and growth factors and hormone action are examples of ROS participation in normal physiology [92,93]. Conversely, a negative role of ROS has been implicated in ageing-related diseases, malignant transformation, atherosclerosis, neurodegenerative diseases, obesity, and diabetes [88,94,95]. Insulin signaling can be also impaired by oxidative stress, but the mechanisms involved are not fully understood. Studies have been demonstrated that ROS lead to impaired insulin response by inducing IRS serine/threonine phosphorylation, decreasing GLUT4 gene transcription, and decreasing mitochondrial activity [37,96].

Chronic elevation in plasma lipids levels and excessive intramyocellular fatty acid disposal are characterized by increased ROS and reactive nitrogen species (RNS) production [15,97,98]. Diet-induced obese mice have an increased expression of inducible nitric oxide synthase (iNOS) and RNS generation in skeletal muscle such effects being associated with impaired insulin sensitivity [15,99], since mice with iNOS disruption in muscle are protected from insulin resistance induced by obesity [100,101]. Diabetic patients present elevated ROS production in endothelial cells through NADPH oxidase activation, a mechanism mediated by PKC [102]. NADPH-oxidase complex is also found in skeletal muscles, raising the possibility that a similar mechanism occurs under elevated FA availability. Our group recently demonstrated that palmitate induces superoxide production in cultured skeletal muscle cells via NADPH oxidase activation, at least in part [98]. Some evidence also links xanthine oxidase (XO) as a source of increased ROS generation in diabetes and obesity. XO protein and activity is found increased in muscle arterioles and livers from animal models of type 1 diabetes and diet-induced obesity [88,103,104].

Animals treated either with high-fat diet or oxidant drugs such as buthionine sulfoximine (BSO), an inhibitor of gluthatione synthase, have increased skeletal muscle ROS production, oxidative stress and are insulin-resistant [105,106]. On the other hand, animals food restricted or treated with antioxidant drugs such as N-acetyl-cysteine (NAC), lipoic acid, vitamin E, and taurine, have reduced oxidative stress and improved insulin sensitivity [106-108]. In addition, mice overexpressing SOD2 have decreased ROS levels, improved hepatic insulin sensitivity, normalization of blood glucose and insulin levels, and reduced activation of cellular stress signalling pathways [109]. These data suggest that mitochondrial ROS is important for the development of insulin resistance.

The involvement of oxidative stress in insulin resistance was also observed in studies performed in myocytes. In L6 muscle cells, H_2_O_2 _reduced insulin-stimulated glucose uptake and glutathione content, effects that were prevented by preincubation with the antioxidant lipoic acid [110]. Rat soleus muscle exposed to nitric oxide (NO) donors have decreased insulin-stimulated glucose uptake and glycogen synthesis, effects that were associated with reduced insulin-stimulated phosphorylation of IR, IRS-1, and Akt [15].

Since mitochondria is the main site of ROS production in skeletal muscle, mitochondrion DNA, protein and lipids components are exposed to high levels of these metabolites suffering structural modification and damage which at long term can result in impaired function of this organelle [111,112].

Mitochondrial dysfunction or reduced mitochondrial biogenesis and density can lead to a decrease in mitochondrial fatty acids oxidation, which results in increased levels of fatty acil-CoA and DAG that can activate stress-related Ser/Thr kinase activity and inhibit glucose transport, as reviewed by Lowell and Shulman [113].

In the case of stress-activated kinases, oxidative stress also contributes to impair insulin signaling by increased uncoupling protein-2 (UCP-2) activity. When these proteins are activated, it results in a heat generation that does not contribute to ATP production [114,115]. UCP-2 negatively regulates glucose-stimulated insulin secretion by reducing the ATP production, which is key to provide energy for almost all cellular processes [113]. In addition, UCP-2^-/- ^mice demonstrate enhanced insulin secretory capacity after a high-fat diet due to improved β-cell functions in a type 2 diabetes animal model [116].

Mitochondrial uncoupling is a powerful tool to control ROS formation and consequently to preserve mitochondrial function. It can be hypothesized that fatty-acid induced uncoupling decrease mitochondrial ROS production and thus it can prevent mitochondrial lipotoxicity. In fact, UCP-3 is upregulated using high-fat diets [117,118], fasting [119], etomoxir treatment (which inhibits the mitochondrial fatty acid oxidation) [120,121] and lipid infusion [122], all conditions being associated with excessive lipid accumulation in skeletal muscle. On the other hand, when fat oxidative capacity is improved, like with endurance training [123,124], weight loss [125], or lowering circulatory fatty acids [126,127] there is a decrease in UCP-3. Interestingly, UCPs are activated by fatty acids and/or its peroxidation products, reducing mitochondrial ROS production [128,129]. So, it can be suggested that UCP-3 is involved in the protection against mitochondrial lipotoxicity by decreasing ROS production when activated by FA, as reviewed by Schrauwen *et al. *[130].

In summary, oxidative stress seems to play an important role in mitochondrial dysfunction, which can further exacerbate stress signals and reduce ATP production. The pathways leading to insulin resistance may be synergistic and mitochondrial dysfunction can create a feedback loop, adding to the overall oxidative stress environment [87].

### Impairment of skeletal muscle mitochondrial function by saturated fatty acids

Several studies have shown that mitochondrial content, mitochondrial function, and oxidative capacity are decreased in insulin-resistant obese and type 2 diabetic individuals [131,132] suggesting that mitochondrial dysfunction might play an important role in the pathophysiology of insulin resistance. Corroborating with this hypothesis, impaired mitochondrial function and reduced fatty acid oxidative capacity were found in isolated primary myocytes, isolated rectus abdominal muscle strips and muscle homogenates from insulin-resistant obese and type 2 diabetic patients [26,83,133]. Moreover, mitochondrial density is reduced in insulin-resistant skeletal muscle from children, offspring of people with type 2 diabetes, suggesting that impaired mitochondrial oxidative capacity can be an inherited defect and an early marker for the development of insulin resistance [96]. Some studies have also reported alterations (mutations, polymorphisms, and epigenetics) in the mitochondrial DNA in conditions of insulin resistance, such as obesity, type 2 diabetes, and metabolic syndrome [134-137].

Evidence points for an important role of fatty acids in the genesis of the mitochondrial dysfunction associated with obesity and type 2 *diabetes mellitus*. In this sense, lipid infusion or administration of high-fat diet to health human and rodents were associated with impaired mitochondrial function characterized by a reduction in ATP synthesis, oxygen consumption and oxidative phosphorylation [16,75,138,139]. These findings were corroborated by *in vitro *studies in which treatment of cultured skeletal muscle cells with palmitic acid increased ROS production, impaired fatty acid oxidation and decreased PGC-1 expression [103,104,140,141]. Studies from our group demonstrated that saturated fatty acids directly induces mitochondrial dysfunction in C2C12 skeletal muscle cells, as evidenced by reduced ATP synthesis and mitochondrial polarization [5].

### Mitochondrial dysfunction plays a central role in the fatty acid-induced insulin resistance

**Figure 1:**
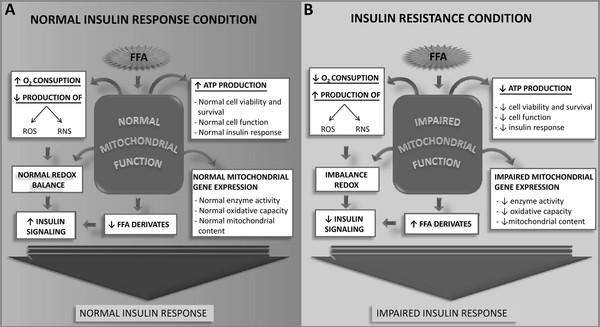
**Role of mitochondria in the insulin resistance induced by free fatty acids (FFA)**. In the normal condition (**A**), mitochondrial function is normal and FFA are rapidly metabolized with low reactive oxygen species (ROS) production and without accumulation of lipid metabolites; normal insulin response is preserved in this condition. In pathological condition (**B**), the excess of plasma FFA levels induces high FFA uptake into the cell, modulating negatively the expression of genes related to mitochondrial biogenesis and oxidative capacity, and into the mitochondrion, increasing the electron flux through to electron transport chain and, consequently, ROS and RNS production. As a result, mitochondrial biogenesis and function are impaired, decreasing mitochondrial mass and oxidative capacity, leading to abnormal intracellular accumulation of lipid metabolites and ROS and RNS, which activate some protein kinases involved in the phosphorylation of IRS-1 on threonine and serine residues. When phosphorylated in threonine and serine residues, IRS-1 is not phosphorylated on tyrosine residues, preventing activation of downstream signalling pathways by insulin. In addition, RNS increases IRS-1 nitrosilation, resulting in high degradation of these protein, which can contribute to impaired insulin response

As discussed above, several mechanisms have been proposed to explain the insulin resistance induced by saturated fatty acids. All these mechanisms operate in coordinated, integrated manner linking fatty acids availability to skeletal muscle insulin resistance. To account for this multifactorial characteristic of saturated fatty acid actions, we propose herein an integrative model centered on mitochondrial dysfunction as an important factor in the genesis of insulin resistance induced by fatty acids (Figure 1).

In physiological conditions, fatty acids are normally and rapidly oxidized with low ROS production, little intracellular lipid accumulation and preservation of insulin sensitivity (Figure 1A). In pathological conditions, chronic elevation in circulating fatty acid levels reduces the expression of genes involved in mitochondrial biogenesis oxidative capacity and increase production of ROS, impairing mitochondrial biogenesis and function (Figure 1B). As a consequence, oxidative capacity is impaired and mitochondrial mass is reduced, increasing still further ROS production, leading to accumulation of fatty acid-derived metabolites such as diacylglycerol and ceramides.

**Figure 2:**
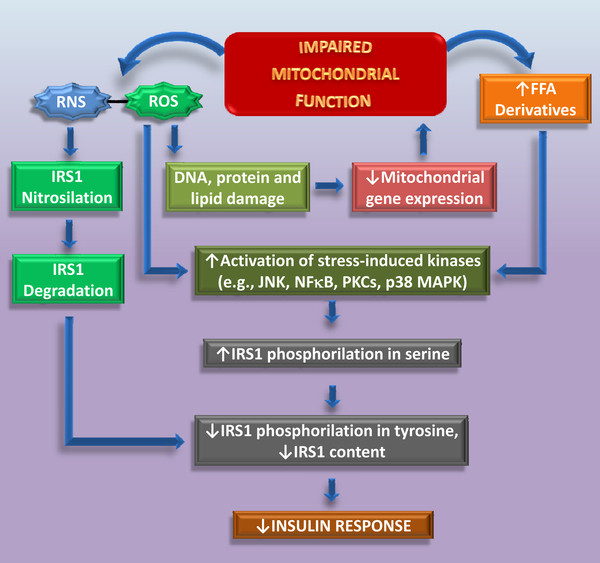
**Mechanisms underlying insulin resistance by impaired mitochondrial function**.

ROS and lipid metabolites have been positively associated with insulin resistance and activation of several kinases, such as NFκB, p38 MAP kinase, JNK, and some novel and atypical PKC isoforms, as PKC-ζ and -ε, in skeletal muscle [27,36,50,141-144]. These kinases impair the insulin signaling pathway by inducing serine/threonine phosphorylation in IRS-1. Under this condition, insulin-stimulated tyrosine phosphorylation of IRS-1 is inhibited, impairing activation of downstream signaling pathways and decreasing glucose uptake and metabolism in response to the hormone [5,6,145,146] (Figure 2).

### Concluding remarks

We discussed herein the mechanisms involved in insulin resistance in skeletal muscle cells induced by high availability of FFA. Several mechanisms have been proposed, such as Randle cycle, inhibition of insulin signaling pathway, regulation of gene expression and enzymatic activities, increase in ROS and RNS production, and impairment in mitochondrial function. Although numerous studies have been performed in order to investigate each one of these mechanisms, the conclusive proposition has not been defined yet. Evidence suggests that these mechanisms are not exclusive and there are several data in the literature pointing out that more than one mechanism is involved in the insulin resistance induced by FFA. We proposed herein a unifying hypothesis that places the importance of mitochondria in the establishment of FFA-induced insulin resistance.

## Competing interests

The authors declare that they have no competing interests.

## Authors' contributions

ARM, RTN, and RHL participated of the acquisition, analysis, and interpretation of the data from literature. RG, MAV, and WTF organized the structure of the manuscript and had substantial contribution to the conception and design. MFCB and LRS were involved in the drafting of the manuscript and figures. SMH and RC critically revised the text and figures for the final version of the manuscript. All authors read and approved the final manuscript.
